# Itraconazole Loaded Biosurfactin Micelles with Enhanced Antifungal Activity: Fabrication, Evaluation and Molecular Simulation

**DOI:** 10.3390/antibiotics12101550

**Published:** 2023-10-19

**Authors:** Faisal Usman, Mudassir Farooq, Tanveer A. Wani, Hassan Ahmad, Ibrahim Javed, Mazhar Iqbal, Fatima Akbar Sheikh, Farhan Siddique, Seema Zargar, Saleh Sheikh

**Affiliations:** 1 Department of Pharmaceutics, Faculty of Pharmacy, Bahauddin Zakariya University, Multan 66000, Pakistan; saleh_dms@outlook.com; 2 Department of Manufacturing Pharmacy, Faculty of Pharmacy, Mahidol University, Bangkok 10400, Thailand; mudassir.far@student.mahidol.ac.th; 3 Department of Pharmaceutical Chemistry, College of Pharmacy, King Saud University, P.O. Box 2457, Riyadh 11451, Saudi Arabia; 4 Faculty of Pharmaceutical Sciences, University of Central Punjab, 1-Khayaban.e. Jinnah Road, Johar Town, Lahore 54000, Pakistan; h.ahmad@ucp.edu.pk; 5 Center for Pharmaceutical Innovation, Clinical and Health Sciences, The University of South Australia, North Terrace, Adelaide 5000, Australia; ibrahim.javed@unisa.edu.au; 6 Health Biotechnology Division, National Institute for Biotechnology and Genetic Engineering (NIBGE), Faisalabad 44000, Pakistan; migondal@nibge.org; 7 College of Pharmacy, Niazi Medical and Dental College, Sargodha 40100, Pakistan; fatimatahir303@gmail.com; 8 Department of Pharmaceutical Chemistry, Faculty of Pharmacy, Bahauddin Zakariya University, Multan 66000, Pakistan; drfarhansiddique@bzu.edu.pk; 9 Department of Biochemistry, College of Sciences, King Saud University, P.O. Box 22452, Riyadh 11451, Saudi Arabia; szargar@ksu.edu.sa

**Keywords:** itraconazole, micelles, surfactin A, hemolysis, molecular simulation

## Abstract

Itraconazole (ITZ) is a broad-spectrum antifungal for superficial subcutaneous and systemic fungal infections. This study aimed to enhance the antifungal activity of ITZ using surfactin A (SA), a cyclic lipopeptide produced by the SA-producing *Bacillus* strain NH-100, possessing strong antifungal activity. SA was extracted, and ITZ-loaded SA micelles formulations were prepared via a single-pot rinsing method and characterized by particle size, zeta potential, and infrared spectroscopy. In vitro dissolution at pH 1.2, as well as hemolysis studies, was also carried out. The fabricated formulations were stable and non-spherical in shape, with an average size of about 179 nm, and FTIR spectra depicted no chemical interaction among formulation components. ITZ-loaded micelles showed decreased hemolysis activity in comparison to pure ITZ. The drug released followed the Korsmeyer–Peppas model, having R^2^ 0.98 with the diffusion release mechanism. In an acidic buffer, drug release of all prepared formulations was in the range of 73–89% in 2 h. The molecular simulation showed the outstanding binding and stability profile of the ITZ-SA complex. The aromatic ring of the ITZ mediates a π-alkyl contact with a side chain in the SA. It can be concluded that ITZ-loaded micelles, owing to significant enhanced antifungal activity up to 6-fold due to the synergistic effect of SA, can be a promising drug delivery platform for delivery of poorly soluble ITZ.

## 1. Introduction

Healthcare practitioners face a significant issue as the prevalence of fungal diseases rises at a dangerous rate. Fungi entering the human body can cause fungal infections. Mycoses or fungal infections were confined to particular regions in the past, but they are now widespread. Individuals’ vulnerability to mycosis has increased due to genetic diversity within innate or adaptive immunity genes, infections like HIV and cancer. Around a billion individuals have fungal skin, hair, and nail infections. Of these, many millions have mucosal candidiasis, and more than 0.15 billion have severe fungal illnesses that can significantly impact a person’s life and even be deadly in some circumstances. A wide range of serious fungal infections, including coccidioidomycosis, aspergillosis, sporotrichosis, blastomycosis, cryptococcosis, para-coccidioidomycosis, histoplasmosis, and disseminated Penicillium marneffei infections, are treated clinically with itraconazole (ITZ), a broad-spectrum antifungal agent [1]. ITZ has been utilized by clinicians to treat candidemia and has demonstrated effectiveness comparable to fluconazole for treating oral Candida infections in AIDS [2]. ITZ is a lipophilic, crystalline compound with limited water solubility and a molecular weight of approximately 705.6 g/mol. It exhibits a pKa of about 3.7 and a logP (partition coefficient) value of around 6.8, reflecting its hydrophobic nature, which can impact its oral absorption and formulation challenges [3]. ITZ shows dose-dependent toxicity, particularly when used at high doses or for extended periods. This can lead to adverse effects such as liver toxicity, gastrointestinal issues, and cardiovascular complications, including QT interval prolongation, which can be life-threatening. Furthermore, ITZ can have significant drug interactions, making it challenging to use alongside other drugs that patients may require. ITZ’s pharmacokinetic profile, including its dependence on gastric acidity for absorption, can lead to inconsistent drug levels in some patients, potentially impacting treatment outcomes [4]. Various attempts have been made to reduce dose-dependent toxicity of ITZ by forming nanoparticles [5], cyclodextrin complexation [6] and transferosomes [7]. After an extensive study to increase ITZ’s bioavailability and enhanced activity at lower doses, a new oral formulation of ITZ-loaded micelles has been created employing biosurfactants, such as surfactin A (SA). Biosurfactants, commonly recognized as surface active agents derived from biological sources, are synthesized by microorganisms such as bacteria, yeasts, and fungi either externally or in association with their cell wall. These compounds have significant surface activity and have numerous potentials uses in the biomedical domain owing to their antibacterial, antifungal, antiviral, hemolytic, antithrombotic, anticancer, cAMP phosphodiesterase inhibition and anti-HIV properties [8]. SA is a cyclic lipopeptide biosurfactant generated by microorganisms. SA from plant growth-promoting rhizobacteria (PGPR), such as *Bacillus subtilis* species, were used in this investigation. It has been suggested that SA possesses pharmacological effects such as antifungal and antibacterial activities [9], as well as anti-inflammatory [10] and thrombolytic characteristics [11]. Due to SA’s biodegradability and low toxicity, biosurfactants are preferable to synthetic surfactants. SA is soluble in both water and other polar solvents. SA’s amphiphilic nature makes it simple to utilize in various nano formulations, including liposomes, micelles, nanoparticles and microemulsions [12]. 

Micelles are vital in drug delivery as they provide a nano-sized carrier system, enabling the solubilization and effective delivery of hydrophobic drugs to target sites, improving their therapeutic efficacy while minimizing the side effects. The critical micelle concentration (CMC) is a key parameter in micelle formation as it represents the concentration at which micelles form, ensuring efficient drug encapsulation for hydrophobic drugs, while below the CMC, they remain as individual molecules, maintaining stability and preventing premature drug release [13]. It also shows the maximum concentration of ionic strength, pH and temperature-dependent biosurfactant monomers in water. The SA has a low CMC value that reduces surface tension more effectively at the lowest concentration. Even at low concentrations, the high surface action of SA decreases the surface tension of water by around 2.5 times [14]. These micelles enclose the drug molecules, increasing their stability and solubility. The hydrophobic part of the micelle makes it easier to penetrate the fungal cell membranes, while the hydrophilic surface of the micelle interacts with the aqueous environment [15].

The present study explores the development and evaluation of ITZ-loaded biosurfactin micelles. By harnessing the unique properties of SA, we aim to enhance the therapeutic potential of ITZ at a lower dose against severe fungal infections, offering a promising avenue for improved patient care in the face of this escalating global health concern.

## 2. Results

### 2.1. Characterization of ITZ-Loaded Micelles

#### 2.1.1. % Entrapment Efficiency EE (%) and % Drug Loading

The EE (%) of all fabricated formulations (ITZ1, ITZ2, ITZ3, ITZ4 and ITZ5) was 71.08 ± 3.66 to 84.31 ± 2.54%, while % drug loading was in the range of 81.41 ± 3.40 to 89.15 ± 2.28%. The high EE (%) and % drug loading were observed in ITZ2 and ITZ3 formulations, as shown in Table 1.

#### 2.1.2. Particle Size, Polydispersity Index and Zeta Potential

The particles size of the fabricated formulation was 131.7 ± 3.61 to 241.4 ± 1.93 nm, as depicted in Table 1. All these formulations were in nanometer range [16]. All the prepared micelles formulations of ITZ had negative zeta potential values and lie in the range of −2.96 ± 1.56 to −22.19 ± 4.24 mV. The optimized ITZ2 formulation has a zeta potential of −2.96 ± 1.56 mV with PDI 0.28 ± 1.36, showing micelles are monodispersed. The negative value was increased as the concentration of SA rises.

#### 2.1.3. Transmission Electron Microscopy (TEM) 

**Figure 1:**
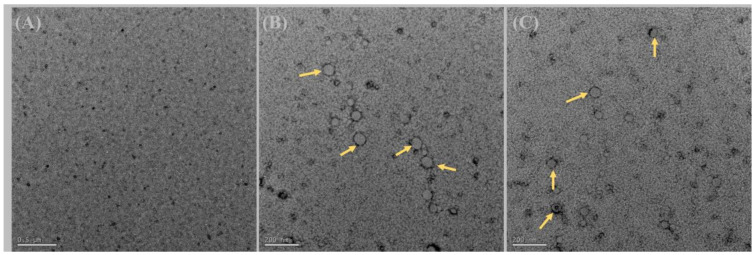
Transmission electron microscopic images of surfactin A (**A**), blank micelles (**B**) and ITZ-loaded micelle formulation, ITZ2 (**C**). The micelles were spherical as shown by yellow arrows.

The TEM image of SA (Figure 1A) depicted small, irregularly shaped aggregates resembling debris. Within these aggregates, small pores are discernible, suggesting variations in the electron density of SA molecules. TEM images of blank (Figure 1B) and loaded micelles (Figure 1C) showed that the micelles were spherical as shown by yellow arrows [17].

#### 2.1.4. Fourier Transform Infrared Spectroscopy (FTIR)

**Figure 2:**
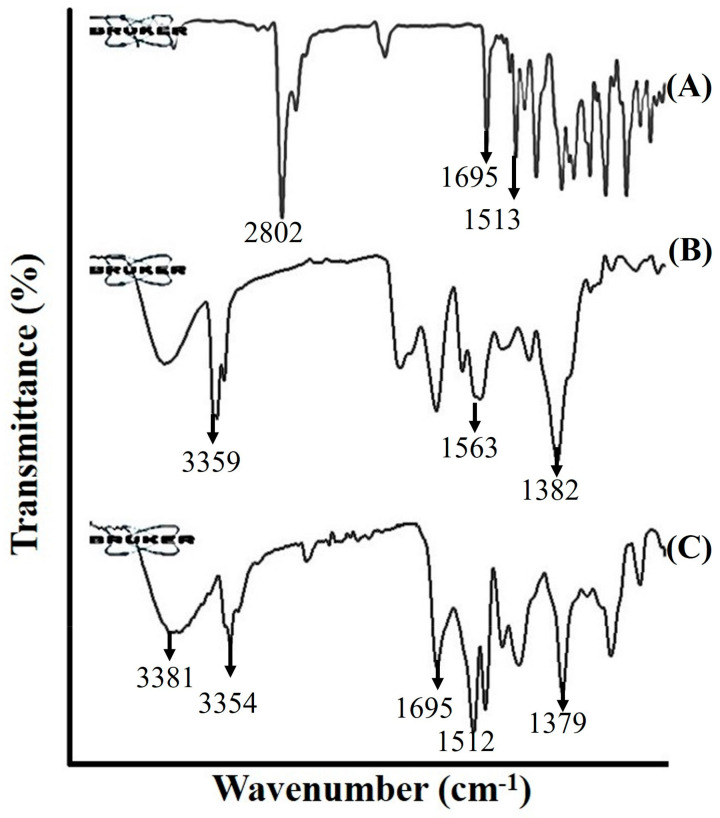
FTIR spectra of Itraconazole (**A**), surfactin A (**B**) and itraconazole-loaded micelles formulation, ITZ2 (**C**).

Figure 2 displays the infrared (IR) spectra of ITZ, SA and ITZ2 formulations. The FT-IR spectra of pure ITZ exhibit distinct peaks at 1695 cm^–1^ and 1513 cm^–1^, as reported in previous findings by Van et al. [18]. The observed spectral peaks at 1695 cm^–1^ and 1513 cm^–1^ correspond to the vibrational modes associated with the C=O functional group and C-H bonds, respectively. The existence of an aliphatic (CH_2_) group in the ITZ spectra was indicated by the prominent peak observed at 2802 cm^–1^. The SA exhibited pronounced absorption bands, which suggest the existence of a peptide component at 3359 cm^–1^, arising from the stretching mode of the N-H bond. The stretching mode of a CO-N bond was found at 1563 cm^–1^. The identification of an aliphatic chain was determined by the observation of C-H stretching modes at a wavenumber of 1382 cm^–1^. The obtained findings provided the confirmation of the existence of SA, which comprises aliphatic and peptide-like components.

The FTIR spectrum of the ITZ micelle formulation ITZ2 revealed a signal corresponding to SA at 3359 cm^–1^. However, this peak is shown to be slightly moved to a higher wavelength of 3381 cm^–1^, indicating a change in the NH- bond stretching compared to pure SA. The disappearance of the peaks at 1382 cm^–1^ and 1563 cm^–1^, which were attributed to CH- bond stretching and CO-N, respectively, can be attributed to the interaction between ITZ and SA. This formulation has a distinctive peak at 1512 cm^–1^, which was similar to the peak observed in the pure drug at 1513 cm^–1^. The peak resulting from the C=O bond in the pure drug ITZ exhibited a shift towards a higher wavelength, namely at 1708 cm^–1^, as compared to its original position at 1695 cm^–1^. The observed phenomenon can likely be attributed to the presence of van der Waals forces [19,20].

### 2.2. In Vitro Drug Release

**Figure 3:**
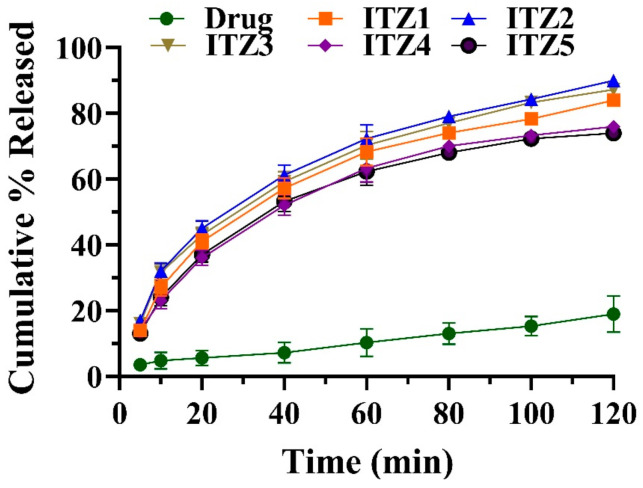
Cumulative % released of fabricated formulation (ITZI to ITZ5) and pure itraconazole (drug) (mean ± SD, *n* = 3).

In an acidic buffer of pH 1.2, drug release of all prepared formulations was found in the range of 89.98 ± 1.81% to 73.24 ± 2.35% in 2 h. By contrast, the drug released was 18.76 ± 2.11% in the control formulation containing pure ITZ. ITZ is a weakly basic drug, its solubility was decreased in an acidic environment, resulting in a lowered dissolution of pure ITZ, suggesting the decreased absorption of ITZ in the stomach. In comparison to the micellar formulation (ITZ1–ITZ2), the cumulative % released was enhanced in an acidic environment. The maximum drug cumulative % released was observed in the ITZ2 formulation containing 1:2 of ITZ and SA. The cumulative % released of pure ITZ and fabricated micelles formulations are presented in Figure 3. 

### 2.3. Drug Release Kinetics

Table 2 displays the release kinetics analysis of pure ITZ and fabricated optimized micelles formulation, ITZ2. The pure ITZ and ITZ2 release data did not well match the zero-order, first-order or Higuchi models. The Peppas equation has been used to describe the drug release process having R^2^ for ITZ 0.96 and for ITZ2, 0.98. Korsmeyer–Peppa’s hypothesis states that drug release follows the Fickian diffusion process. The values of the release exponent “n” are shown in Table 2, indicating a Fickian release mechanism of ITZ2 in which the drug release was regulated by diffusion [21].

### 2.4. Antimicrobial Activity

A significant reduction in minimum inhibitory concentration (MIC) and minimum fungicidal concentration (MFC) was reported in the formulation of ITZ2. The ITZ2 formulation exhibited superior antifungal activity compared to the control formulation (CF) containing pure ITZ. No evidence of fungal susceptibility was seen in the blank sample. The minimum inhibitory concentration (MIC_50_) values of ITZ against *Candida albicans*, *Aspergillus fumigatus* and *Aspergillus niger* were 0.25 ± 0.19 µg/mL, 0.014 ± 0.93 µg/mL and 0.11 ± 0.092 µg/mL, respectively. The MFC of ITZ against *Aspergillus fumigatus*, *Aspergillus niger* and *Candida albicans* are illustrated in Table 3.

### 2.5. Stability

No discernible alterations in the visual characteristics of all formulations were noted throughout a six-month storage period at room temperature (25 ± 2 °C/60% RH) and elevated temperature (40 ± 2 °C). A slight reduction in the percentage of EE (%), particle size, PDI and ZP was detected, as depicted in Table 4.

### 2.6. In Vitro Hemolysis 

**Figure 4:**
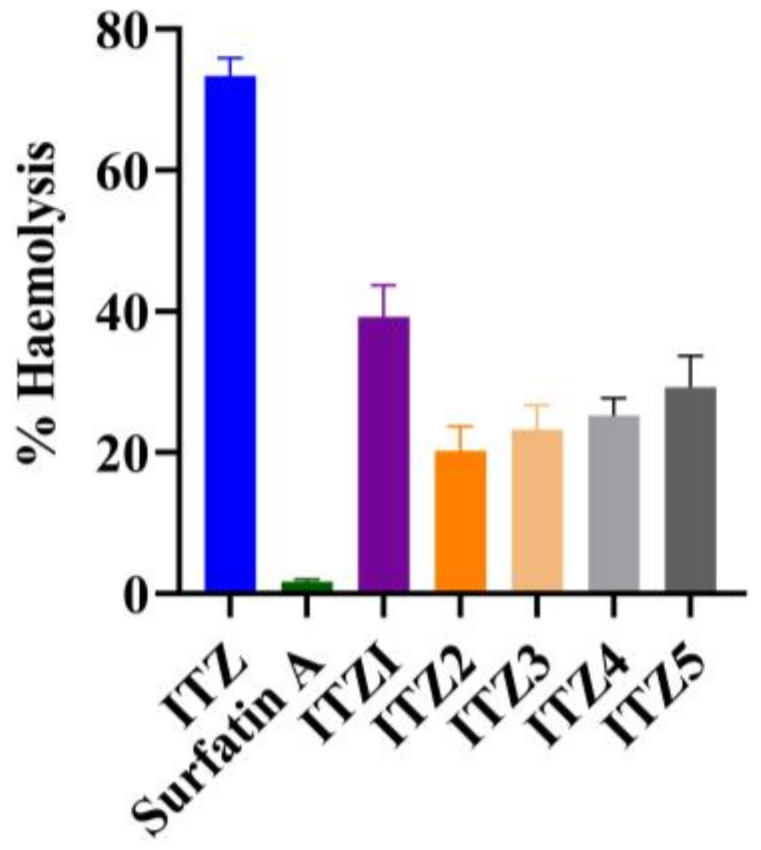
% hemolysis of Itraconazole (ITZ), surfactin A and itraconazole formulations, ITZ1 to ITZ2 (mean ± SD, *n* = 3).

The hemolytic assay is a widely employed method for the characterization of biosurfactant biocompatibility, serving as the sole approach utilized for this purpose [15]. In order to assess the blood safety profile or hemocompatibility of the formulated substances, the impact of ITZ, SA and ITZ micelles formulations on the hemolysis of RBCs was examined subsequent to the incubation of micelles with blood. The formulation of ITZ2 micelles had a lower degree of hemolytic activity, 20 ± 0.93% containing 10 µg/mL of ITZ and 20 µg/mL of SA, as illustrated in Figure 4. The SA showed hemolytic activity of 1.9 ± 0.34%. Similar findings were also reported by Sarwar A. et al. (2018) [22]. The hemolytic activity of SA was less than 2%, which was safe to be used for drug delivery applications. In contrast, the ITZ solution resulted in far more pronounced hemolysis. At 10 µg/mL of ITZ examined, the observed tendency for hemolysis was 73 ± 1.7%. This finding was also reported by Sherief Essa et al. (2013) [23]. Overall, the formulations of ITZ3, ITZ4 and ITZ5 suggest that as we further increased the amount of SA in the formulation from 30 µg/mL to 50 µg/mL, the hemolytic activity increased while ITZ1 also showed higher hemolytic activity as compared to ITZ2 to ITZ5, because at lower concentrations, SA did not show any significant effect. The findings of this study suggest that the micellar formulation containing ITZ and SA effectively reduced the hemolytic activity associated with ITZ. In other words, it can be inferred that the SA micelles loaded with ITZ exhibited a greater compatibility with blood cells compared to free ITZ. 

### 2.7. Molecular Docking

**Figure 5:**
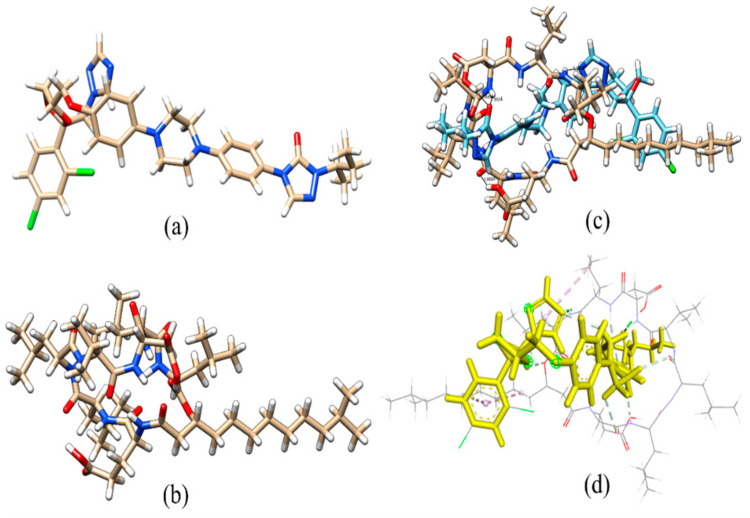
Optimized structure of ITZ (**a**), the optimized structure of surfactin (**b**), optimized complex ITZ-SFN (**c**) and interaction sites with gray lines of ITZ (yellow) and surfactin A (**d**).

**Figure 6:**
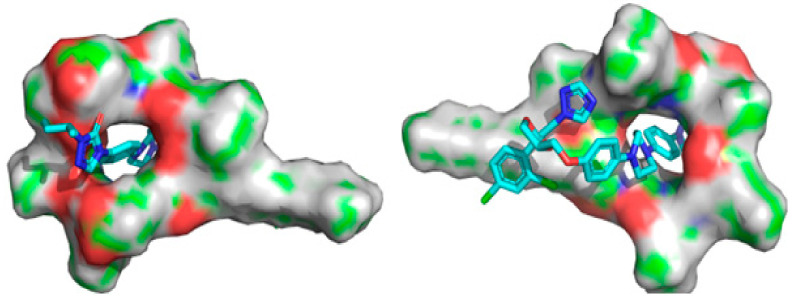
Binding active pocket site for ITZ with surfactin. Front pose (**left**) and back pose (**right**). Cyan color structure showing ITZ and surface showing surfactin.

The ITZ structure comprises triazolone, a linker and dioxolan, with a triazole region. The anchoring step of connecting with surfactants involves these three locations of ITZ. The molecular interactions between ITZ and SA that led to the development of a stable complex are shown in Figure 5. As can be seen in Table 5, the complex is stabilized by several interactions, including eight hydrogen bonds between the ITZ and SA molecules, with binding links between hydrogen, nitrogen and oxygen sites and side chains at distances of 2.28, 1.87, 1.69, 2.81, 2.80, 2.59, 2.56 and 2.46 Å. Within ITZ, the triazole moiety created one hydrogen bond, while the hydroxyl moiety formed the other. As seen in Figure 6, this connection acts as a stability anchor that guides the construction of the ITZ and SA complex. At 3.22, 4.61, 4.45, and 4.84 Å, the aromatic ring of ITZ mediates a π-alkyl contact with a side chain in SA. An outstanding binding and stability profile was also seen for the ITZ–SA complex, with an interaction energy of −39.577 kcal/mol. The enhanced solubility, stability and antifungal activity of the ITZ-loaded SA micelles are all concluded by these several outcomes involving binding interaction energy, hydrogen bonding, alkyl contacts and the active site pocket. Molecular docking studies revealed that the enhanced solubility, stability and antifungal activity of the ITZ-loaded micelles are all concluded by several outcomes involving binding interaction energy, hydrogen bonding, alkyl contacts and the active site pocket.

## 3. Discussion

This research aimed to improve ITZ’s solubility and increase its antifungal activity. Biosurfactants are becoming a popular alternative to chemical surfactants. Previously, Zhang and colleagues published a study on the oral administration of insulin via SA [24]. It was known that the concentration of SA affected the average particle size and PDI of the micelles. Also, micelles with low PDI were spread out evenly, and PDI values of less than 0.3 were thought to be acceptable but also associated with non-covalent binding [22]. The amphiphilic framework of SA enhanced the repulsive force between micelles, and the electrostatic repulsion increased with a higher surface charge and a thicker diffuse layer, which increased the zeta potential of ITZ5. Also, the changes in micelles particle size may be caused by the saddle framework of SA, which consists of both hydrophilic and hydrophobic parts and gives strong interfacial activity [23]. Also, as the amount of SA increased, the hydrophobic interactions between the micelles became stronger. They led to a denser hydrophobic core and an increase in non-covalent binding, which led to a rise in ITZ EE (%) and % drug loading [24]. Due to strong repulsive forces between micelles, the larger zeta-potential micelles were more likely to be stable. Electrostatic repulsion between micelles increased ITZ’s interaction and spatial repulsion, which prevented the aggregation of micelles when exposed to a bad atmosphere [25].

The release of a hydrophobic drug from SA-based micelles depends on several critical factors, including the concentration of the biosurfactant used, micelle size and structure, drug-biosurfactant interactions, drug loading, micelle stability, the solubility of the drug in the micellar core and pH conditions. The drug release of all prepared formulations was found to be higher in the acidic buffer of pH 1.2. The % drug release was low when we increased the weight of SA from 90 to 225 mg. For instance, ITZ5 containing 225 mg SA showed only a 4.3% increase in the dissolution rate. The marginal increase in drug release suggested that a saturation point may have been reached, limiting its ability to significantly enhance the drug dissolution. Similarly, the % drug release showed a downward trend as the quantity of SA decreased from 90 mg to 45 mg. So, the cumulative % drug released was highly dependent on the quantity of SA used in micelles formation. The almost twenty-fold increase in the cumulative % drug released rate may be due to the increased surface area exposed to the dissolution media and the utilization of SA in the creation of micelles. As the concentration of SA rises, more SA molecules aggregate and organize themselves into larger micellar structures. However, this increase in micelle size can have a direct impact on the % drug release. Larger micelles possess a smaller surface area-to-volume ratio, which reduces the exposure of the hydrophobic drug to the surrounding medium. This decreased surface area hinders the efficient dissolution and release of the ITZ, resulting in a lower % drug release rate. Therefore, the increase in particle size as surfactin A concentration rises leads to reduced drug release due to the reduced accessibility of the drug to the dissolution medium within the larger micellar structures [25]. The drug release kinetics followed the Korsmeyer–Peppa hypothesis as the release exponent “n” of ITZ2 was 0.42, indicating a Fickian release mechanism in which the drug release was regulated by diffusion [21]. Similar findings of biosurfactant-based formulations were also reported by Wadhawan, A. et al. (2022) [26].

The formulations were stable at room temperature and accelerated temperature and humidity, with just a minor change in appearance that might be due to drug leakage from the micelles’ core. Pure SA and ITZ showed no hemolysis activity at a concentration below 40 µM. The correlation between molecular docking and FTIR results for the ITZ-SA complex revealed a compelling connection between molecular interactions and spectral changes. Molecular docking analyses indicated that the complex was stabilized by multiple hydrogen bonds, π-alkyl contacts and a strong interaction energy. These interactions were supported by shifts and disappearances in the FTIR spectra of the complex, suggesting the formation of hydrogen bonds and van der Waals forces. Notably, shifts in peaks related to C=O and NH-bond stretching indicated changes in bond characteristics due to the interaction. Overall, the FTIR findings provided experimental evidence of the molecular interactions proposed by docking studies, reinforcing the complex’s stability and the role of specific bonds in its formation. 

The lower hemolytic activity observed in ITZ2 in comparison to pure ITZ can be explained by several key factors. Firstly, ITZ2’s micellar structure provides a protective shield for ITZ, encapsulating it within a hydrophobic core surrounded by a hydrophilic biosurfactant shell, limiting direct contact between the drug and red blood cells (RBCs). This configuration reduces the likelihood of ITZ causing disruption to RBC membranes, minimizing hemolysis. Additionally, the micelle formulation enhanced the ITZ solubility and stability, preventing the formation of aggregates or crystals that could potentially damage RBCs. The presence of SA may also contribute to membrane stabilization. These combined factors lead to a safer blood compatibility profile for ITZ2, as demonstrated by its lower hemolytic activity, while free ITZ lacks these protective mechanisms, resulting in higher hemolytic potential [27]. The in vitro antifungal study indicated that SA boosted the ITZ’s antifungal efficacy against *Candida Albicans*, *Aspergillus fumigatus* and *Aspergillus niger* due to the synergic effect of SA and micelles. Their synergism may be due to SA’s influence on the cell membrane. 

## 4. Materials and Methods

### 4.1. Materials

ITZ was generously donated by Ferozsons Laboratories, Nowshera, Pakistan. 1,4 Dioxane, methanol, chloroform, dimethyl sulfoxide (DMSO) and methyl propane sulfonic acid were purchased from Sigma Aldrich^®^ Biochemie GmbH, Hamburg, Germany. Double distilled water was obtained from the distillation unit installed in the laboratory, faculty of pharmacy, Bahauddin Zakariya University, Multan, and SA was extracted from bacterial lipopeptides.

### 4.2. Extraction and Purification of Surfactin A

Extraction and purification of SA were carried out using the method described previously by Ambrin Sarwar et al. in 2018 [22]. Briefly, the lipopeptides were identified using RMB7 inoculation. RMB7 was put into a 100 mL Erlenmeyer flask of broth. After being incubated for 36 h in a glass flask at a temperature of up to 28 °C, the culture was centrifuged. Lipopeptides were recovered as residues after the medium’s pH was changed to an acidic pH of 2 using 6M HCL via centrifugation in a 2:1 mixture of methanol and water. Syringe filters were used to filter the recovered lipopeptides, vacuum drying was carried out and they were preserved by resuspending in methanol. In broth medium (Landy broth) for one day, several bacterial strains (NH100), as well as reference strains (FZB-42), were cast. After adjusting the quantity of bacterial strains, a further incubation was carried out by combining 250 mL of the broth with 1 mL of the bacterial strains. After 4 days of incubation, the pH of the medium was regulated using HCL, then a group of cells was added. After a day of refrigeration, the collected cells underwent a second centrifugation. Cells were lyophilized, combined with ethanol to dissolve them and filtered. Acetonitrile, water and trifluoroacetic acid (80:20:0.05) were employed as the mobile phases via high-performance liquid chromatography process to evaluate surfactant A by injecting 20 µL of the sample at λ_max_ 214 nm at a flow rate of 1 mL/min with C18 column of 250 × 4.6 mm (Perkin Elmer, Seer Green, Beaconsfield, IA, USA). Standard SA (Sigma Aldrich, St. Louis, MO, United States) produced the calibration curve. Thus, pure SA was produced, which was then dried under nitrogen and preserved for later examination.

### 4.3. Preparation of ITZ Micelles

**Figure 7:**
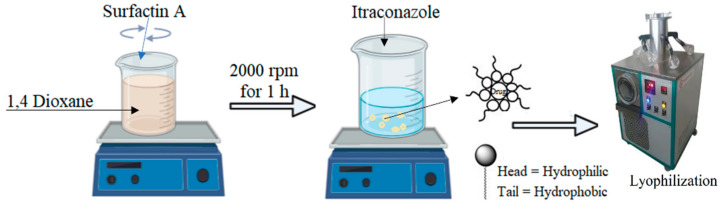
The schematic representation of fabrication of ITZ-loaded micelles.

Micelles were produced using the single-pot rinsing technique described by Usman F, 2017 [28]. A total of 45 mg of SA was dissolved in 1,4 dioxane solvent and stirred continuously for 20 min, and then, 45 mg of ITZ was steadily introduced with continual stirring at 2000 rpm for 1 h at room temperature, as illustrated in Figure 7. Similarly, different formulations with differing ITZ and SA ratios, 1:2, 1:3, 1:4 and 1:5, were developed. The formulations were lyophilized and reconstituted in distilled water to obtain the required concentration for further analysis. A similar procedure was used to create blank micelles devoid of ITZ. Table 6 shows the composition of ITZ micelles.

### 4.4. Characterization of Micelles

#### 4.4.1. % Entrapment Efficiency or EE (%) and Drug Loading

ITZ-loaded micelles were dispersed in dimethyl sulfoxide (DMSO) to cause micelle breakdown and release of the ITZ to calculate the percentage of ITZ loading. The EE (%) was measured from the ratio of the weight of ITZ content present in the micelles to the total ITZ content. The ITZ-loaded micellar solution was placed in a centrifuge tube and centrifuged at 10,000 rpm for 10 min to measure EE (%). The amount of ITZ in the filtrate was analyzed by a UV-vis spectrophotometer at 262 nm (Lambda 850+, Perkin Elmer, USA) [29].

#### 4.4.2. Particle Size, Polydispersity Index and Zeta Potential

The particle size, polydispersity index (PDI) and zeta potential (ZP) were measured using Zetasizer (Nano ZS, ZEN3600, Malvern, UK). Samples were diluted with filtered double distilled water (1:100) and were filled in a disposable cuvette for analysis at constant temperature (25 °C). Since the samples contained DMSO (10%), the refractive index and viscosity parameters were adjusted accordingly in the Malvern Zetasizer™, v7.12 software. Particle size measurements included the intensity mean, z-average, PDI and volume percentage.

#### 4.4.3. Transmission Electron Microscopy (TEM)

The aqueous solution of micelles or surfactant was deposited on a glow-discharged, carbon-coated TEM copper grid and blotted after 2 min. The grid was washed with a drop of water twice and blotted in between the washing. The grid was stained with 1% uranyl acetate solution for 30 s, washed with water and then air dried. The TEM grids were imaged under a Hitachi HT7700 (Nishi-shimbashi, Minato Tokyo, Japan) electron microscope at an operating voltage of 80 kV [30].

#### 4.4.4. Fourier Transform Infrared Spectroscopy (FTIR)

To verify the interaction of ITZ in SA micelles, the FTIR spectrophotometer (Bruker, Tensor 27 series, Rheinstetten, Germany) was used to record the spectra of pure drug and ITZ-loaded micelles. With a resolution of 4 cm^−1^, the frequency range was chosen between 600 cm^−1^ and 4000 cm^−1^ [31,32]. 

### 4.5. In Vitro Drug Release Study

A drug release study of control formulation (containing pure ITZ) and micelle preparations containing ITZ was carried out at 0.1M HCL buffer of pH 1.2 for 2 h. Micelles formulations containing ITZ were accurately weighed and filled in a dialysis membrane. The sample was suspended in the dissolving media at 75 rpm and 37 ± 0.5 °C. At regular intervals, a 3 mL aliquot was removed and replaced with a fresh medium. The withdrawn sample was filtered and appropriately diluted before being measured in a spectrophotometer for ITZ absorbance at λ_max_ 262 nm.

### 4.6. Drug Release Kinetics

The drug release kinetics were studied by incorporating data into the zero-order, first-order, Higuchi and Korsmeyer–Peppas models using Equations (1)–(4).

(1) $$F_{\mathrm{rel}}={\;K}_{o}t$$

(2) $$l_{n}\left(1-F\right)=-K_{f}{t\;K}_{o}t$$

(3) $$F_{\mathrm{init}}=K_{H}t$$

(4) $$\frac{M_{t}}{M}=K_{p}t_{n}$$

F_init_ and F_rel_ are initial fractions and release fractions of the drug in time ‘t’. The zero-order, first-order, Higuchi and Korsmeyer–Peppas release constants are represented by the letters K_o_, K_f_, K_H_ and K_P_. The units M, M_t_ and n stand for the mass of water absorbed at equilibrium, the amount of water absorbed over time (t) and the release exponent, respectively [33].

### 4.7. In Vitro Antimicrobial Activity against Fungi

Clinical and Laboratory Standards Institute (CLSI) guidelines for broth microdilution susceptibility testing were used to test the antifungal activity of blank formulation (placebo), ITZ2 and control formulation containing pure ITZ in DMSO and ITZ2. The samples were diluted twice in RPMI 1640 medium buffered at pH 7 with 0.165 molL^−1^ morpholine propane sulfonic acid, and then they were put in 96-well microtiter plates. Each microdilution well contained 100 μL of serial dilutions of the compounds and 100 μL of fungal suspensions to give a final inoculum size of 0.5 to 2.5 × 10^3^ and 2.4 × 10^4^ colony-forming units per mL for *Candida albicans* and *Aspergillus niger* and *Aspergillus fumigatus*, respectively, and sample concentrations were in the 0.48 to 250 μg/mL range. Visual examination indicated the minimum inhibitory concentrations (MICs) after incubation at 35 °C for 48 h for *Candida albicans*. MIC is the lowest chemical concentration that suppresses visible fungal growth in microdilution wells. After the MIC determination, 100 μL aliquots from each well with no apparent growth and the growth control were subcultured onto Sabouraud dextrose agar plates and incubated at 35 °C for 48 h for *Candida albicans*. The minimal fungicidal concentration (MFC) is the lowest drug concentration that precluded observable fungal colony formation on the plate [34].

### 4.8. Stability Studies

The prepared ITZ-loaded micelles lyophilized powder formulations were subjected to accelerated stability testing. As per ICH requirements for accelerated stability investigations, samples were stored in the stability chamber for 6 months at 40 °C and 75% relative humidity. The samples were evaluated for in vitro dissolution rate and drug content after 1 month [35].

### 4.9. In Vitro Hemolysis

The hemolytic assay is a widely employed method for the characterization of biosurfactant production, serving as the sole approach utilized for this purpose [36]. A 10 mL sample of heparinized human blood was recently acquired and utilized to assess the hemolytic activity of different formulations. The blood sample was subjected to centrifugation at a speed of 3000 rpm for a duration of 15 min. Subsequently, the red blood cells (RBCs) were isolated from the liquid portion above the sediment, known as the supernatant. The RBCs should be washed three times using a PBS of pH 7.4 in order to eliminate any debris and serum proteins. Following this, the RBCs should be diluted by a factor of 200. A negative control sample was prepared by combining RBCs with buffer solution in a 1:1 molar ratio, whereas a positive control sample was prepared using RBCs treated with a 5% *v*/*v* solution of Triton X100. The investigation focused on assessing the hemolytic activity of pure ITZ and ITZ2 formulations, at a concentration of 10 µg/mL of ITZ and 10 µg/mL of SA. The incubation phase lasted for a duration of 24 h at a temperature of 37 °C, accompanied by stirring at a rate of 200 rpm. Subsequently, the samples were placed on ice for a duration of 5 min in order to halt the process of hemolysis, following which they were subjected to centrifugation at a speed of 16,000 rpm for a period of 30 s. Subsequently, the liquid portion obtained from each sample was carefully transferred into individual wells of a 96-well plate, and the optical density was quantified at a wavelength of 541 nm [37,38,39]. The % hemolysis was measured using Equation (5).

(5) $$\%\;\mathrm{Hemolysis}=\left(\frac{\mathrm{Sample}\;\mathrm{test}-\mathrm{Negative}\;\mathrm{control}\;\mathrm{sample}}{\;\mathrm{positive}\;\mathrm{control}\;\mathrm{sample}-\mathrm{Negative}\;\mathrm{control}\;\mathrm{sample}}\right)\times 100$$

### 4.10. Molecular Docking

The structure of ITZ was obtained in the SMILE format from the drug bank web server. The structure of SA was obtained from PubChem [40]. Initial optimization of ITZ and SA was carried out using B3LYP functional [41,42] and 6-311+G(d,p) basis set in a gas phase, always including the D3 correction for dispersion [43,44]. By taking optimized structures of ITZ and SA species, optimization for ground-state ITZ-loaded SA micelles structure was carried out. All the calculations were performed using the Gaussian16 suite. 

### 4.11. Statistical Analysis

The data were statistically examined to establish their level of significance. Unless otherwise specified, data are reported as mean and standard deviation (SD) from at least five samples. The data were analyzed using analysis of variance (ANOVA) and other appropriate statistical parameters as needed. The statistical significance level was set at *p* < 0.05.

## 5. Conclusions

We have successfully developed a novel ITZ-loaded micelles formulation using a biosurfactant, demonstrating promising results in enhancing ITZ’s antifungal activity while reducing dosage requirements and potential adverse effects. The micelles formulation offers improved stability and solubility, facilitating targeted drug delivery to fungal infection sites. SA’s amphiphilicity enables the efficient penetration of fungal cell membranes and interaction with the aqueous environment, creating a dual-action mechanism that might mitigate the risk of drug resistance. However, despite these encouraging findings, further research and in vivo studies are essential to validate the efficacy and safety of ITZ-loaded micelles. Our study has provided insights into the potential of biosurfactant-based micelles as a promising platform for enhancing the therapeutic properties of antifungal drugs, offering hope for more effective treatments.

## Figures and Tables

**Table 1:** Particle size, zeta potential, polydispersity index, EE (%) and % drug loading of ITZ micelle formulations, ITZ1 to ITZ5 (mean ± SD, *n* = 3).

Formulations Code	Particle Size (nm)	Zeta Potential (mV)	Polydispersity Index	% Entrapment Efficiency	% Drug Loading
ITZ1	220.1 ± 2.43	−3.13 ± 1.12	0.22 ± 1.65	73.05 ± 1.23	84.45 ± 3.40
ITZ2	131.7 ± 3.61	−2.96 ± 1.56	0.28 ± 1.36	84.31 ± 2.54	88.06 ± 1.76
ITZ3	187.3 ± 4.31	−7.49 ± 2.76	0.21 ± 3.75	83.73 ± 0.94	89.15 ± 2.28
ITZ4	193.5 ± 2.33	−14.36 ± 1.56	0.18 ± 2.81	79.65 ± 2.23	82.23 ± 1.73
ITZ5	241.4 ± 1.93	−22.19 ± 4.24	0.27 ± 3.94	71.08 ± 3.66	81.41 ± 2.07

**Table 2:** Drug release kinetics model of pure itraconazole (ITZ1) and itraconazole micelles formulation (ITZ2).

Formulation Code	Zero-Order Release	First-Order Release	Higuchi Model	Korsmeyer–Peppas Model	
	R^2^	R^2^	R^2^	R^2^	*n*
Pure ITZ	0.88	0.89	0.93	0.96	0.65
ITZ2	0.42	0.93	0.96	0.98	0.42

**Table 3:** Minimum inhibitory concentration (MIC_50_) and minimum fungicidal concentration (MFC) of different fungus strains in µg/mL (mean ± SD, *n* = 3).

Species	Formulations	MIC_50_	MFC
*Aspergillus fumigatus*	BF	-	-
	ITZ2	0.25 ± 0.19	1.23 ± 0.079
	CF	0.46 ± 0.89	1.58 ± 0.098
*Aspergillus niger*	BF	-	-
	ITZ2	0.014 ± 0.93	0.31 ± 0.094
	CF	0.29 ± 0.99	1.82 ± 0.088
*Candida albicans*	BF	-	-
	ITZ2	0.11 ± 0.092	2.32 ± 0.099
	CF	0.12 ± 0.012	4.21 ± 0.016

BF = Blank formulation (placebo); CF = control formulation containing pure itraconazole; and ITZ2 = itraconazole micelles formulation.

**Table 4:** EE (%), particle size, zeta potential and polydispersity index of ITZ loading micelles after 6 months of storage (mean ± SD, *n* = 3).

Formulation Code	ITZ1	ITZ2	ITZ3	ITZ4	ITZ5
EE (%)	72.95 ± 2.14	83.15 ± 1.09	82.56 ± 1.75	77.49 ± 2.38	71.15 ± 1.55
Particle Size	226.1 ± 4.43	133.5 ± 6.61	198.6 ± 4.31	201.2 ± 3.36	247.6 ± 5.26
Polydispersity Index	0.24 ± 1.65	0.29 ± 1.36	0.24 ± 3.75	0.20 ± 2.81	0.33 ± 3.94
Zeta Potential	−3.53 ± 2.1	−2.63 ± 1.7	−7.74 ± 2.4	−13.93 ± 3.1	−29.36 ± 4.2

**Table 5:** Hydrogen bonding, π-bonding, and electrostatic interactions with distances in Angstrom for investigated ITZ–surfactin complex.

Atom Linkage	Distance (Å)	Type of Interaction
H-N	2.28	Hydrogen Bond
H-O	1.87	Hydrogen Bond
H-O	1.69	Hydrogen Bond
H-O	2.81	Hydrogen Bond
H-N	2.80	Hydrogen Bond
N-H	2.58	Hydrogen Bond
N-H	2.56	Hydrogen Bond
H-O	2.46	Hydrogen Bond
H-O	3.22	Pi-Donor Hydrogen Bond
O-H	4.61	Pi-Alkyl
O-H	4.45	Pi-Alkyl
O-H	4.84	Pi-Alkyl

**Table 6:** Formulations with different molar concentrations of ITZ and surfactin A.

Formulation	ITZ (mg)	Surfactin A (mg)
ITZ1	45	45
ITZ2	45	90
ITZ3	45	135
ITZ4	45	180
ITZ5	45	225

## Data Availability

The data presented in this study are available from the authors upon reasonable request.
